# Bacterial Communities from the Copper Mine of Wettelrode (Germany)

**DOI:** 10.3390/life15020204

**Published:** 2025-01-29

**Authors:** J. Michael Köhler, Linda Ehrhardt, P. Mike Günther, Jialan Cao

**Affiliations:** Institute for Micro- und Nanotechnologies/Institute for Chemistry and Biotechnology, Technische University Ilmenau, D-98684 Ilmenau, Germany; linda.ehrhardt@tu-ilmenau.de (L.E.); mike.guenther@tu-ilmenau.de (P.M.G.); jialan.cao@tu-ilmenau.de (J.C.)

**Keywords:** soil bacteria, halophiles, extremophiles, copper mining, salt, sulfate-reducer, sulfur-oxidizer

## Abstract

Bacterial communities from three different sampling sites of a copper mine tunnel were characterized by 16S rRNA sequencing (NGS). A high presence of halophilic bacteria was confirmed by comparison with literature data and with reference samples from other highly salt-exposed soils. Among others, high read numbers of *Gracilimonas*, *Kangiella*, *Limibacillus*, *Marinobacter*, *Woseia*, and uncultivated strains of *Actinomarinales*, *Gammaproteobacterium AT-s16*, *Actinobacteria 0319-7L14*, and *Thiotrichaceae* were found. The community in a sample from the surface of the copper seam was significantly different from the community composition of a sample from the mine tunnel floor. The specificity in the appearance and in the abundance of special bacterial types (for example, *Thiogranum*, *Thiohalophilus*, *Sulfuriflexus*, *Sedimenticolaceae*, *Desulfomonile*, *Desulfosporosinus*, and *Cand. Thiobios*) can be partially explained by the different local conditions for sulfur-related metabolisms at the sampling sites.

## 1. Introduction

Soil microbiomes rank among the most complex and most diverse ecosystems on Earth [1]. The viability and resilience of soil microbial communities are essential for the fertility of the soil, their adaptability to environmental changes, and their ecological role regarding macroorganisms such as plants and animals [2]. The composition of soil bacterial communities is influenced by both chemical and physical conditions, on the one hand, and the fate of the related soil in the past, on the other hand. It is evident that the growth of specific bacteria is dependent on the availability of nutrients and essential chemical components, temperature, humidity, soil pH, salt content, and other environmental parameters [3].

Increased salt content in soil is a significant stress factor as many microorganisms cannot tolerate salt concentrations above certain thresholds. However, some microorganisms are adapted to high salt concentrations, thriving in marine environments or even in highly concentrated brines such as salt lakes [4,5]. Salt not only plays an important role in natural ecosystems but is also a crucial cultural resource. Since prehistoric times, salt has been extracted and used primarily for preserving food, such as in the preparation of salted meat, stockfish, and cheese. The exploitation of natural salt deposits, its transportation and processing, and its use in daily life have inevitably resulted in the release of salt into the environment. This release often affects soils with initially low salt content, introducing a form of anthropogenic chemical stress for the affected soil microorganisms.

Elevated salt concentrations in soils can be detected through chemical analyses or by measuring the electrical conductivity of soil material dispersed in water. High salt levels are often observed in areas associated with historical mining activities, where salt-containing materials were brought to the surface [6]. Other examples include saline production sites, ash deposits, and artisanal zones where salt was used in production processes [7]. Indeed, such places show not only an enhanced electrical conductivity of soil materials but also a certain portion of halotolerant or halophile soil bacteria. Recently, we studied soil bacterial communities from an ash deposition site linked to preindustrial saline activities near the historic saline town of Bad Dürrenberg, close to Leipzig, Germany [8]. Similarly, increased soil conductivity was identified in samples from the eastern medieval suburb of Jena (Germany), a region historically associated with high levels of dyeing and tanning activities [9]. In the above-mentioned cases, the impact of changes in the salt content of soil resulted in special soil bacterial compositions due to the salt stress that was caused by former human activities and is still present today. The soil bacterial community responds to human impacts by changing the chemical conditions for bacterial growth and competition. Thus, soil bacteria serve as indicators of past human impacts, representing part of the “environmental memory of the soil”.

In this study, we examine the presence of specific bacterial types in three samples taken from the copper mine of Wettelrode, Germany. This former subsurface mining area is characterized by groundwater with high salinity [10]. We present preliminary results from next-generation sequencing (NGS) analyses of 16S rRNA, highlighting the distinct compositions of three local sub-ground bacterial communities.

## 2. Experimental Setup

The samples were taken during an excursion in a disused copper mine in Wettelrode near Sangerhausen (Germany) in the southern region of the Harz mountains. The copper seam was formed in the Permian formation Zechstein about 260 million years ago. The copper slate has a sulfidic character. In addition, the copper in this ore is accompanied by other metals. The samples were taken directly from the sediment layers using sterile 50 mL centrifuge tubes. Briefly, 0.5× *g* of sediment material was dispersed and shaken in 5 mL of deionized water for pH and conductivity measurements. pH was determined potentiometrically by using a glass microelectrode and measured using a Mettler Toledo pHIon220. The electrical conductivity was measured using a Mettler Toledo Five Go.

The samples came from a ventilation tunnel, the so-called “östliches Wetterflachen” of the copper mine of Wettelrode (Germany), by cutting the copper seam around 300 m in the ground. Sample W2 was taken directly from the copper seam, W3 was taken from the side wall below the copper seam (“Liegendes”) of the tunnel, and W6 was taken from its floor.

The DNA was extracted using DNeasy^®^ PowerSoil^®^ Pro kits (Qiagen, Hilden, Germany). A commercial laboratory thermocycler Edvocycler (Edvotek, Washington, DC, USA) was used for DNA amplification by PCR. The result of each PCR process was evaluated by using gel electrophoresis to apply agarose gels with a solid content of 1%. Both the primary PCR products and the final DNA libraries after pooling the labeled samples were treated in a ProNex^®^ Size-Selective Purification System (Promega, Madison, WI, USA) for purification following the protocol supplied by the manufacturer.

The adaptor primers for Amplicon PCR A519F-Ad (5′ TCGTCGGCAGCGTCAGATGTGTATAAGAGACAGCAGCMGCCGCGGTAA 3′) and Bact_805R-Ad (5′-GTCTCGTGGGCTCGGAGATGTGTATAAGAGACAGGACTACHVGGGTATCTAATC 3′) were supplied by Eurofins Genomics (Ebersberg, Germany). The primers were applied at a concentration of 0.1 nmol/µL. The following components were mixed in the reaction solution (total volume of 50 µL per reaction): 1 µL of DNA isolation eluate, 2 mM MgCl_2_, 200 µM PCR nucleotide mix, 1.25 Units GoTaq G2 Flexi DNA Polymerase, nuclease-free water (all reagents from Promega, Madison, WI, USA), and 1 µmol/l of each primer. The PCR amplification was performed using the following steps: denaturation for 5 min at 94 °C; 30 amplification cycles involving 30 s of denaturation at 94 °C; 30 s of annealing at 50 °C; and 30 s of primer extension at 72 °C. The temperature cycling was terminated with a final primer extension reaction at 72 °C (5 min).

The required eight forward and twelve reverse primers for the index PCR of a 96-sample set were supplied by integrated DNA Technologies (Coralville, IA, USA). The single primers were applied at a concentration of 1.25 pmol/µL. The following composition was used for the index PCR: total volume of 25 µL per reaction; 2.5 µL of Amplicon PCR product, 2.5 mM MgCl_2_, 300 µM PCR nucleotide mix, 0.5 Units GoTaq G2 Flexi DNA Polymerase, nuclease-free water (all reagents from Promega, Madison (USA)), and 125 pmol/mL of each of the two primers of the respective indexing primer combination.

For index primer PCR, the following program settings were applied: denaturation for 3 min at 95 °C; 30 amplification cycles involving 30 s of denaturation at 95 °C; 30 s of annealing at 55 °C; and 30 s of primer extension at 72 °C. The temperature cycling was terminated with a final extension at 72 °C for 5 min.

The fastq files supplied by NGS were converted into the format fasta. In addition, the-quality of ata were was checked by using the open-source platform Galaxy (https://usegalaxy.org/) (accessed on 19 November 2024). The quality of all investigated datasets was characterized by a median quality score and found to be high, indicating a very high quality of data.

The taxonomical assignment was achieved by aligning the contig files to rRNA databases based on the NCBI cloud using the SILVAngs data analysis service (https://ngs.arb-silva.de/silvangs) (accessed on 19 November 2024). This procedure allowed a detailed analysis on the basis of the previously obtained sequencing data, supplying information about the bacterial community of the related sample [11,12,13]. For all analyses, the preset parameter configurations of the SILVAngs database version 138.1 were applied [13]. In principle, the final NGS data allow the assignment of 16S rRNA-related DNA down to the genus level. However, in some cases, the assignment is only possible for higher taxonomical levels, such as families, orders, classes, or phyla. Therefore, the best assigned taxonomical groups determined for a sequence were defined as the “Operational Taxonomical Unit” (OTU). The details of DNA processing, quality check, sequencing, and data analysis are reported in [9].

## 3. Results and Discussions

### 3.1. Sample pH and Conductivity of Samples and Reference Samples

The three samples show similar pH values slightly above neutrality (W2: 7.6; W3: 7.9; andW6: 8.0). Despite their moderate alkalinity, the samples are characterized by extraordinarily high electrical conductivity values (W2: 3.3 mS/cm; W3: 3.7 mS/cm; and W6: 9.9 mS/cm). These values are much higher than typical values from samples of topsoil taken in natural environments, which are typically below 1 mS/cm and often range between about 0.02 and 0.1 mS/cm (compare, for example, Supplementary Table S1).

The high electrical conductivity indicates high salt concentrations in the mine water circulating within the copper mine. The salinity of mine water is mainly determined by NaCl. Four measurements of mine water of the related South Harz field near Sangerhausen revealed Na contents between 97.5 and 106.6 g/L and a chloride content between 152.5 and 187 g/L. All other metal ions showed significantly lower concentrations, for example, between 2.0 and 8.3 g/L for Ca and between 0.05 and 0.1 g/L for Cu [10]. Surfaces such as the bottom, sidewalls, and other exposed areas are in direct contact with this highly saline solution. Consequently, microorganisms inhabiting the wet sediments of the mine tunnels or growing on the sidewalls are expected to exhibit a high tolerance to NaCl.

For reference, six samples were used, which were also marked by high electrical conductivities, including one sample from the draining area of a disused potash salt mine (V75 (Bischofferode, Germany): 2.7 mS/cm and pH: 9.14), one from the draining area of an industrial copper mine tailings dump, Nienstedt (Germany): 4.7 mS/cm and pH: 7.6), and four from an ash deposit of a preindustrial saline (Bad Dürrenberg (Germany); HB61 and HB61-2: 2.1 mS/cm and pH: 8.24; HB62-1 and HB62-2: 2.3 mS/cm and pH: 8.09). For the latter four samples, OTUs that are known to be halophilic or salt-tolerant have been reported [8]. The samples V75 (Bischofferode) and H5 (Nienstedt) were taken from vegetation-free surface soil, whereas the samples HB61-1, HB61-2, HB62-1, and HB62-2 come from buried soil layers and were taken during an archaeological investigation.

### 3.2. Community Composition by Phyla

**Figure 1:**
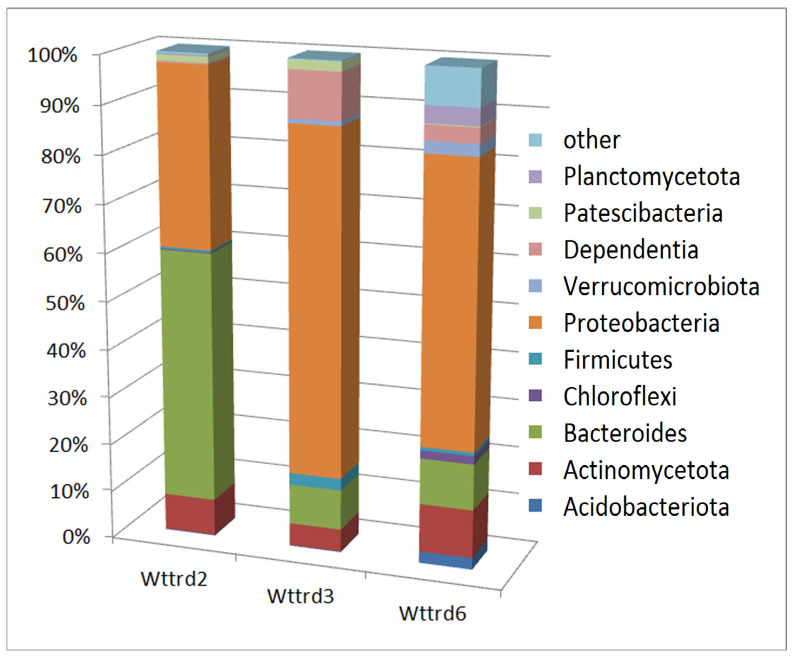
Abundances of bacteria of the three investigated samples by phyla.

**Figure 2:**
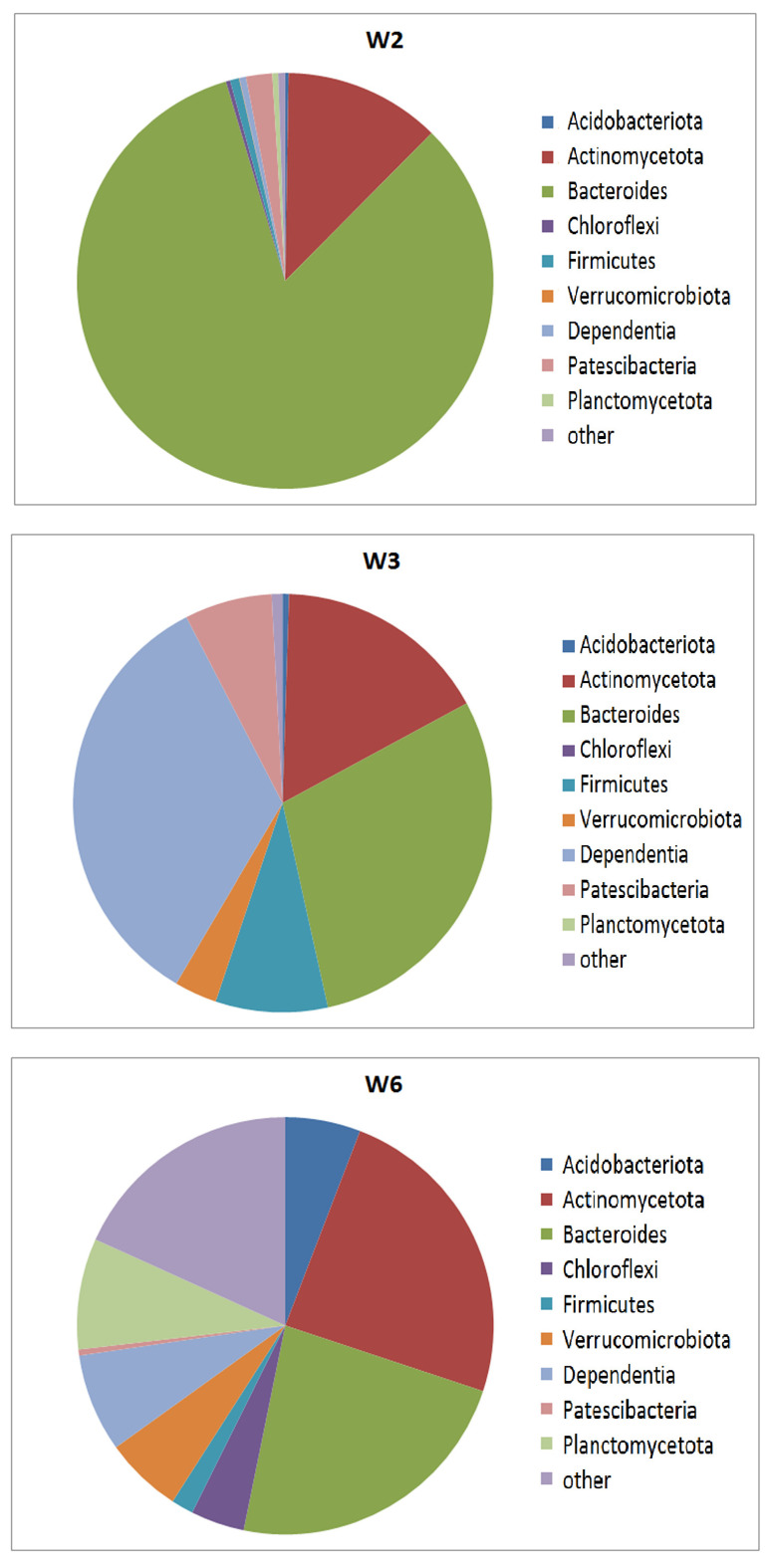
Abundances by phyla without Proteobacteria.

The results of the NGS analyses revealed that the three samples from Wettelrode are dominated by *Proteobacteria* and *Bacteroides*. Figure 1 illustrates the composition of the three samples by phyla, including Proteobacteria. The distribution of abundances by lowest distinguishable taxonomical units is discussed below (Section 3.3). In addition to *Proteobacteria*, the samples are marked by high differences in the abundance of other phyla (for a more detailed illustration, the abundance distribution without Proteobacteria is shown in Figure 2). Sample W2 shows a particularly high presence of *Bacteroides.* A particularly high abundance of *Dependentia* was observed in sample W3. The members of the phylum *Dependentia* are mostly known as parasites of protists. Obviously, they play an important role in aquatic ecosystems, but the knowledge of their ecological functions is limited so far [14]. In addition to *Proteobacteria*, *Dependentia*, and *Bacteroides*, this sample also shows comparatively high abundances of *Patescibacteriota* and *Firmicutes. Patescibacteriota* are frequently found as the main components of groundwater [15]. In comparison to the other samples, sample W6 shows the highest abundances of *Acidobacteriota*, *Actinomycetota*, *Verrucomicrobiota*, *Planctomycetota*, and *Chloroflexi*.

Given the high salinity of the sampling sites, the identified bacteria are likely salt-tolerant or even halophilic. Members of these diverse phyla have been previously reported to include salt-tolerant representatives. To better distinguish the bacterial communities of the three samples, we will now examine their community composition at the OTU level, which, in many cases, corresponds to the genus level.

### 3.3. Community Composition by Genera and OTUs

In addition to the discussion of abundance distribution by phyla (Figure 1 and Figure 2), the abundances of lower taxonomical units (genera or OTUs) supply a better-specified picture of the differences between samples. Despite the NGS-derived read numbers in the same order of magnitude for all three samples (W2: 208,026 reads; W3: 199,915 reads; and W6: 169,938 reads), the numbers of identified OTUs are rather different (more than 10 reads: W2: 109; W3: 63 OTUs; and W6: 222; one read or more: W2: 190; W3: 120; and W6: 457 OTUs). This demonstrates a higher diversity in W6, which corresponds to the abundance distribution of the phyla.

Beyond the phylum-level abundance distribution discussed earlier (Figure 1 and Figure 2), examining the abundances of lower taxonomic units (genera or OTUs) provides a more detailed picture of the differences between the samples. Although the NGS analysis yielded read numbers of a similar magnitude for all three samples (W2: 208,026 reads; W3: 199,915 reads; and W6: 169,938 reads), the number of identified OTUs varied significantly. When considering OTUs with more than 10 reads, the numbers were as follows: W2: 109 OTUs; W3: 63 OTUs; and W6: 222 OTUs. When including all OTUs (one read or more), the numbers increased to W2: 190, W3: 120, and W6: 457. This suggests greater diversity in sample W6, which aligns with the phylum-level abundance distribution without *Proteobacteria* (Figure 2).

The principal difference in the abundance distribution is well-reflected by the Shannon diversity index. Despite the fact that W6 showed a lower total number of reads (169,938) than W2 (208,926 reads) and W3 (199,915 reads), this sample is marked by the highest Shannon diversity index (W2: 2.32; W3: 2.85; and W6: 3.99). Corresponding to these numbers, W6 showed the highest evenness (67.9%) compared to W2 (evenness: 44.2%) and W3 (evenness: 59.5%).

**Figure 3:**
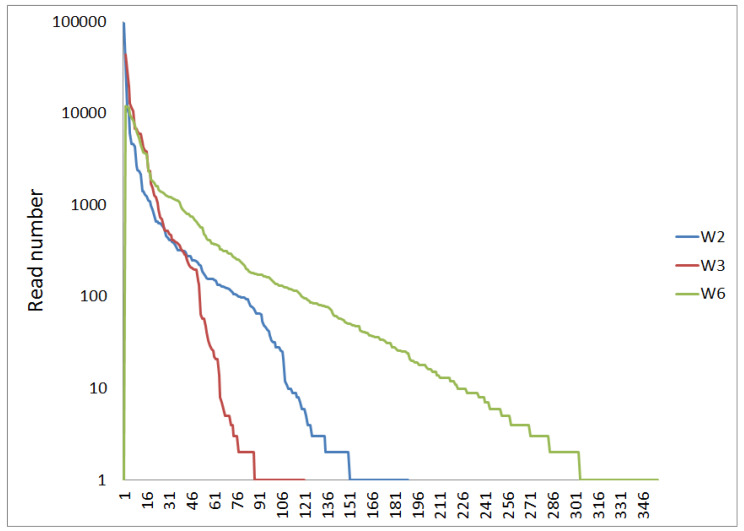
Abundance rank order functions for the three samples.

The difference in the composition of the three bacterial populations is also illustrated by the rank abundance diagrams (Figure 3). While W6 shows regular exponential decay for mediate and low abundances of OTUs, the other two samples (W2 and W3) are marked by a shoulder and a deficit in OTUs, with read numbers below 100. The shoulders in the rank function could indicate a disturbance in the development of the bacterial community [16].

**Figure 4:**
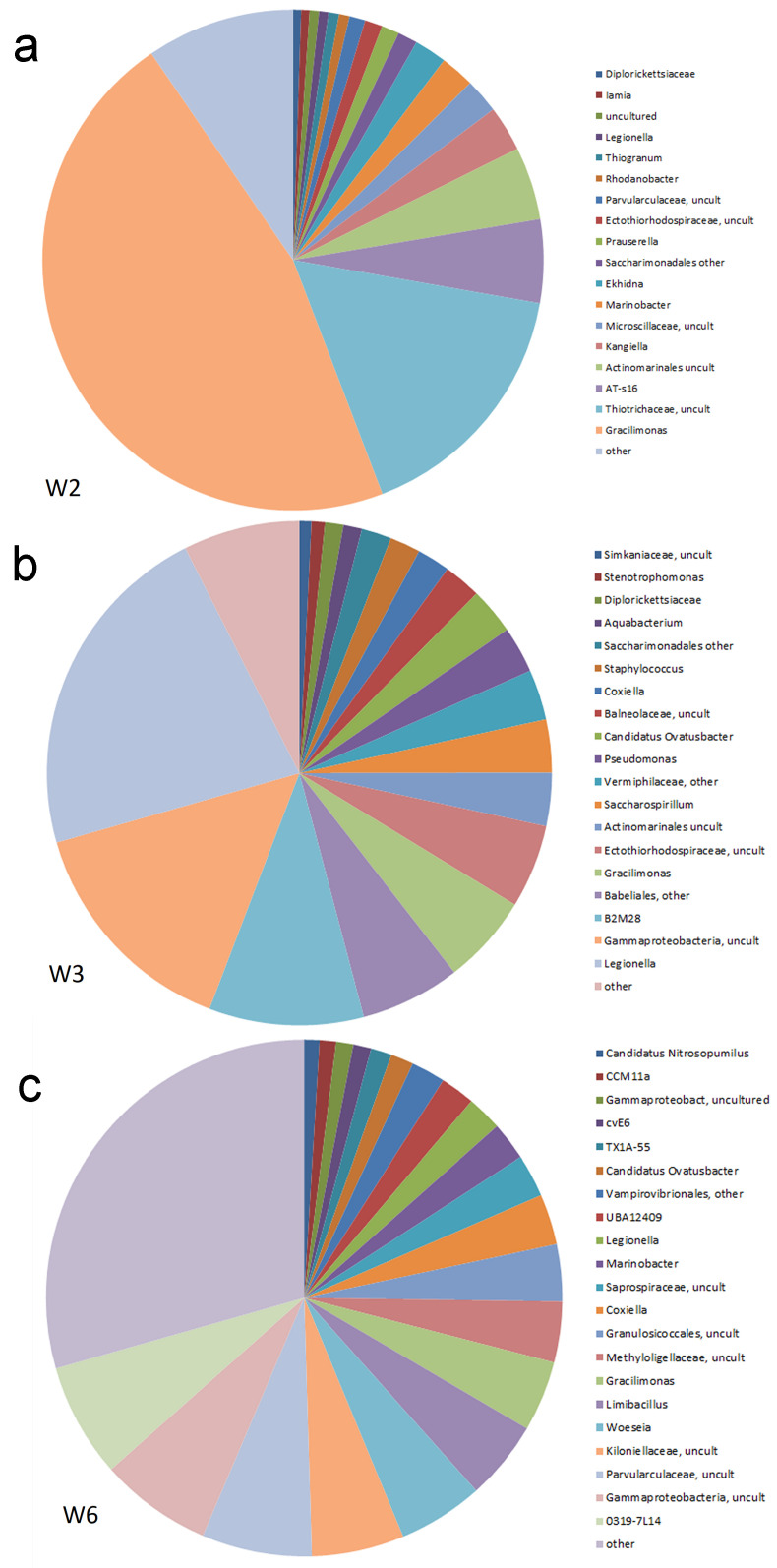
Abundance distribution of OTUs; (**a**) sample W2, (**b**) sample W3, (**c**) sample W6.

Under the OTUs of W2, *Gracilimonas* dominates, with 46% of all reads (Figure 4a). This genus belongs to *Bacteroides*, which explains the dominance of *Bacteroides* among the phyla of W2. *Gracilimonas* was originally found in a maritime environment, is characterized by a strain growing at salt concentrations above 1%, and is able to grow under very high salt concentrations of up to 20% NaCl [17]. *Gracilimonas* is also highly abundant in the other two samples (5.7% of reads in W3 and 4.4% of reads in W6). At least in the case of the samples discussed here, *Gracilimonas* seems to be an important indicator of high salinity. Among the other more abundant genera of W2 are *Kangiella* (2.9%), *Marinobacter* (2.2%), *Ekhidna* (1.5%), and *Thiogranum* (0.7%). These bacteria are also described as salt-tolerant organisms. *Kangiella* was first isolated from tidal sediments of the Yellow Sea and has optimal growth in 2–3% NaCl [18]. *Marinobacter* was found in the Mediterranean Sea and can grow in up to 20% NaCl [19]. *Ekhidna* was first isolated from the southern Pacific Ocean. It shows optimal growth at a NaCl content between 3 and 5% [20]. *Thiogranum* was first described as a bacterium from a deep-sea hydrothermal field growing at a salt concentration of 3% NaCl [21].

In sample W3, *Legionella*, uncultured *Gammaproteobacteria*, and the *Gammaproteobacteria group B2M28* represented nearly 50% of all reads (Figure 4b), which also explains the dominance of *Gammaproteobacteria*.

The diagram of OTU abundances clearly reflects the higher diversity in sample W6 (Figure 4c). In this sample, strongly dominant OTUs, such as in W2 and W3, are absent. In addition to some groups of non-cultivated bacteria and *Gracilimonas*, several other genera with well-known salt tolerance were found. This concerns *Woeseia* (4.5% of reads), *Limibacillus* (4.2%), and *Marinobacter* (2.0%). *Woeseia* was isolated from coastal sediments and grows in salt concentrations of up to 8% [22]. *Limibacillus* was isolated from reclaimed land and showed optimal growth at 2% NaCl [23].

There are also several OTUs that have been found exclusively in one of the three samples. Thus, W2 supplied *Rhodanobacter* (1413 reads), *Marinoscillum* (566 reads), *Sphingorhabdus* (397 reads), *Algoriphagus* (387 reads), *Aquibacter* (307 reads), *Oleiagrimonas* (250 reads), *Microvirga* (157 reads), *Achromobacter* (152 reads), *Angustibacter* (14 reads), *Imperialibacter* (116 reads), *Snuella* (106 reads), *Thermicanus* (99 reads), *Zeaxanthinibacter* (98 reads), *Defluviimonas* (79 reads), *Constrictibacter* (42 reads), *Janibacter* (28 reads), and *Muricauda* (25 reads). A considerable part of these genera was described from marine or otherwise salty environments, including *Marinoscillum*, which tolerates up to 7% sea salt [24], *Algoriphagus* [25], *Aquibacter* [26], *Snuella* [27], *Zeaxanthinibacter* [28], *Defluviimonas* [29], and *Muricauda* [30]. The genus *Rhodanobacter* was first described as a Lindan-degrading species [31]. *Sphingorhabdus* was found in arctic soil, tolerating up to 5% NaCl [32]. *Oleiagriomonas* was isolated from an oil field [33]. *Imperialibacter* was found in Permian groundwater [34], which is of particular interest here because the mine tunnel of Wettelrode cuts directly into Permian sediments. *Thermicanus* was described as a thermophilic bacterium with optimal growth at 60 °C [35]. The majority of these exclusive community components confirm their adaptation in salty environments.

In W3, compared to the others, the following OTUs appear to be exclusive: *Salinisphaera* (470 reads), *Shigella* (412 reads), *Exiguobacterium* (393 reads), *Hyphobacterium* (226 reads), and *Gardnerella* (199 reads). *Salinisphaera* and *Hyphobacterium* are described from marine environments [36,37] too, but *Garderella* and *Shigella* are known as pathogens [38].

Sample W6 had exclusive OTUs too, including *Desulfomonile* (656 reads), *Enhydrobacter* (328 reads), *Nitrospina* (182 reads), *Bythopirellula* (139 reads), *Mariprofundus* (97 reads), *Parachlamydia* (85 reads), *Waddlia* (81 reads), *Leptonema* (81 reads), *Pelagibus* (71 reads), *Cerasicoccus* (57 reads), *Amphiplicatus* (49 reads), *Erythrobacter* (34 reads), *Marinimicrobium* (25 reads), *Filifactor* (25 reads), and *Sulfuriflexus* (24 reads). *Nitrospina* [39], *Bythopirellula* [40], *Mariprofundus* [41], and *Marinimicrobium* [42] are also described from marine environments. *Amphiplicatus* is a thermophilic bacterium isolated from a hot spring and has been shown to tolerate up to 7.5% NaCl [43]. The sulfur-oxidizing genus *Sulfuriflexus* was first described from the sediment of a brackish lake [44]. These community components clearly show the importance of salt-adapted bacteria in the sediment on the floor of the mine tunnel.

### 3.4. Comparison to Reference Samples of Enhanced Salt Content

**Figure 5:**
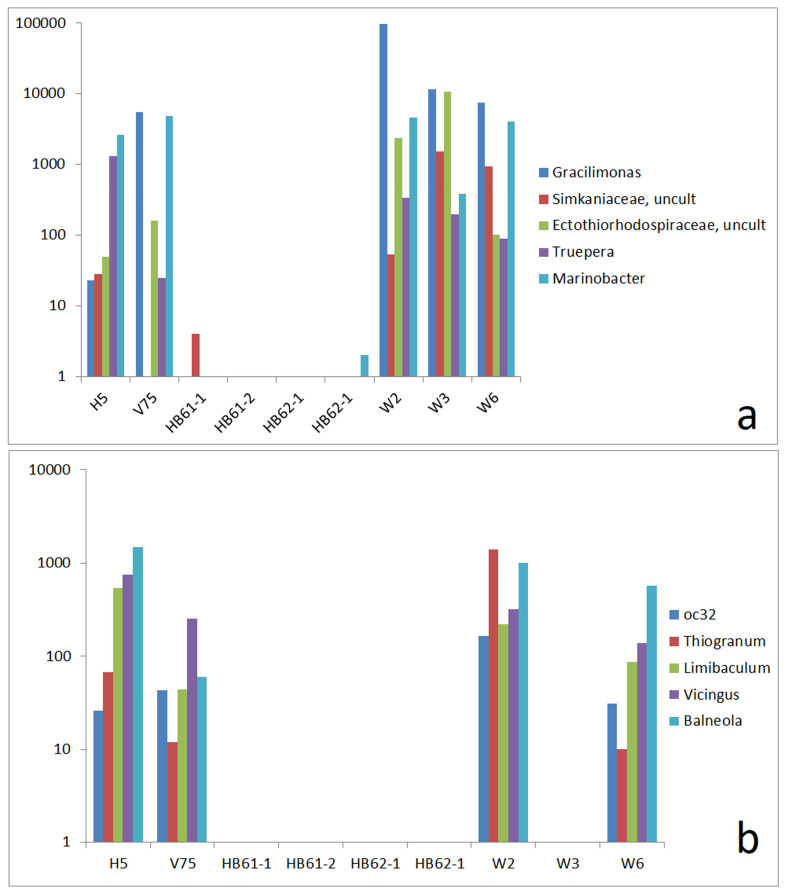
Comparison of absolute read numbers of a set of select OTUs; abundance of OTUs in reference samples and samples W2, W3, and W6 by read numbers: (**a**) OTUs that are present in H5, V74, W2, W3, and W6 and mostly absent in HB61-1, HB61-2, HB62-1, and HB62-2 and (**b**) OTUs that are present in H5, V74, W2, and W6 but mostly absent in HB61-1, HB61-2, HB62-1, HB62-2, and W3.

In addition to the general presence of halophilic types, the reference samples from highly electrically conductive soils exhibit some specific combinations of components, which are also found in the samples from the copper mine. The high abundance (more than 1000 reads) of *Gracilimonas*, *Marinobacter*, and *Marinoscillum* in sample W2 and sample V75 (reference sample from the efflux of the potash mine salt deposit) is particularly striking. A group of OTUs mostly related to salty environments was found in H5 and the three copper mine samples too (Figure 5a). Among them, *Truepera* was described as a thermophilic bacterium tolerating up to 6% NaCl [45]. The efflux of the copper mine tailings dump (sample H5) showed a similar presence of these OTUs, whereas most of them were absent in the highly electrically conductive soils of the deposits of the saline ashes (HB61-1, HB61-2, HB62-1, and HB62-2).

The relationship between the reference samples V75 and H5 and the copper mine sample W3 is less pronounced. However, the OTUs that indicate similarities between the communities of these reference samples and W3 are almost completely missing in the saline ash soil samples.

Samples W2, W6, H5, and V75 are also marked by a common group of five mostly mediate abundant OTUs, which are absent in W3 and in the saline ash deposit soils (Figure 5b). The five bacteria (*Thiogranum*, *Nitrosomonadaceae oc32*, *Vicingus*, *Balneola*, and *Limibaculum*) have been found in marine environments [20,46,47,48] or are otherwise described as halophilic [48]. The group could be understood as a fingerprint feature of salt-tolerant OTUs connecting samples H5, V75, W2, and W3 and excluding the other samples.

A certain exception is formed by a fingerprint group consisting of *Methyloceanibacter*, *Amphiplicatus*, and *NRL2* and OTUs marked as belonging to the families *Criblamydiaceae* and *Fodinicurvataceae*. This group appears in W6 as well as in HB62-1 and HB62-2. The samples HB61-1 and HB61-2 showed a combination of *Methyloceanibacter* and *NRL2* too.

**Figure 6:**
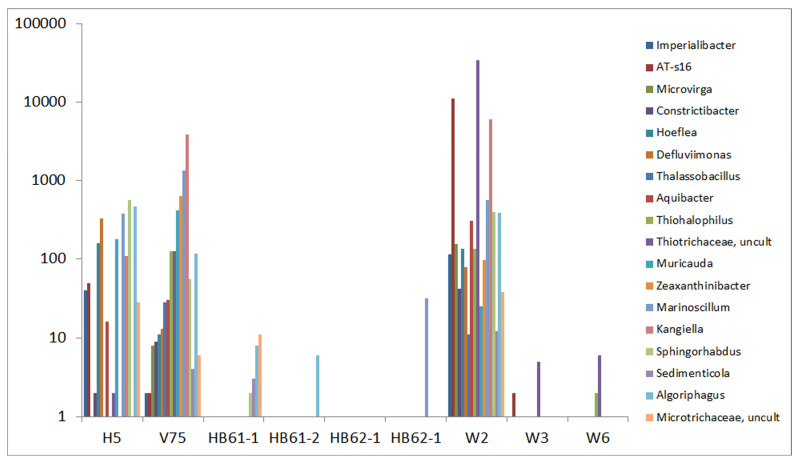
Comparison of absolute read numbers of a set of select OTUs; abundance of OTUs in reference samples and samples W2, W3, and W6; and common abundance of OTUs in reference sample V75 and sample W2.

A special fingerprint-like pattern including 18 OTUs was also observed for the pairing of the copper seam sample W2 with the reference sample V75. The related OTUs were partially present in H5 too but were mostly absent in the other samples (Figure 6). In this group, several salt-related bacteria were also observed, including *Kangiella*, *Algoriphagus*, *Aquibacter*, *Zeaxanthinibacter*, *Defluviimonas*, *Muricauda*, *Sphingorhabdus*, *Thiohalophilus*, *Hoeflea* [49], and *Thalassobacillus* [50].

**Figure 7:**
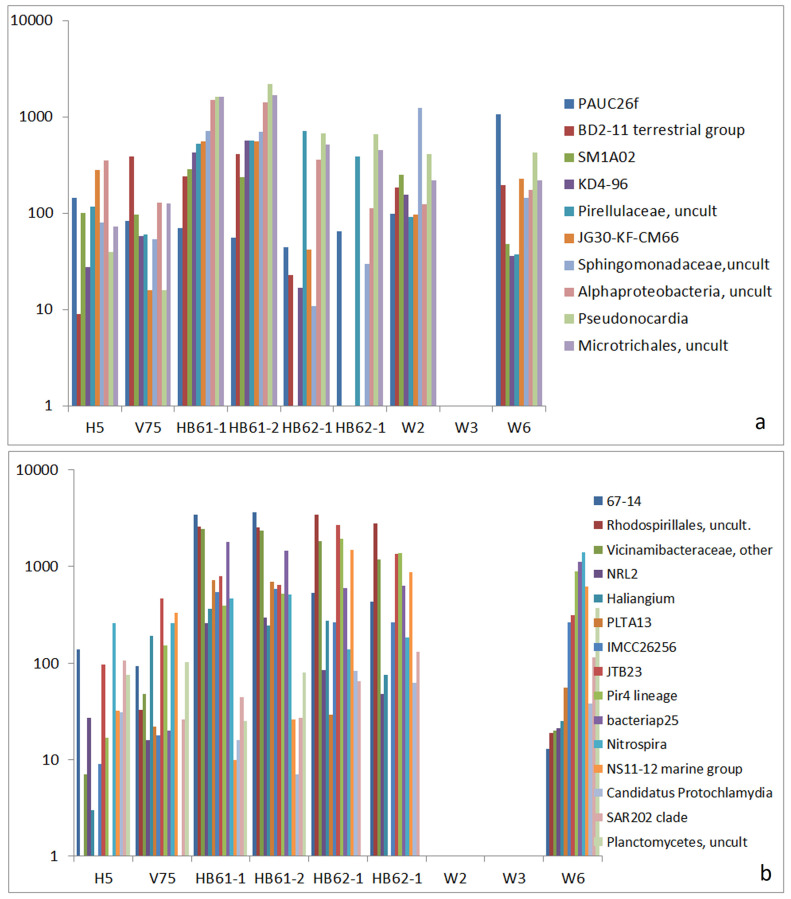
Comparison of absolute read numbers of a set of select OTUs; abundance of OTUs in reference samples and samples W2, W3, and W6 and abundance of OTUs in all reference samples and samples W2 and W6 by read numbers: (**a**) OTUs that are present in W2 and W6 and (**b**) OTUs that are present in W6 only.

**Figure 8:**
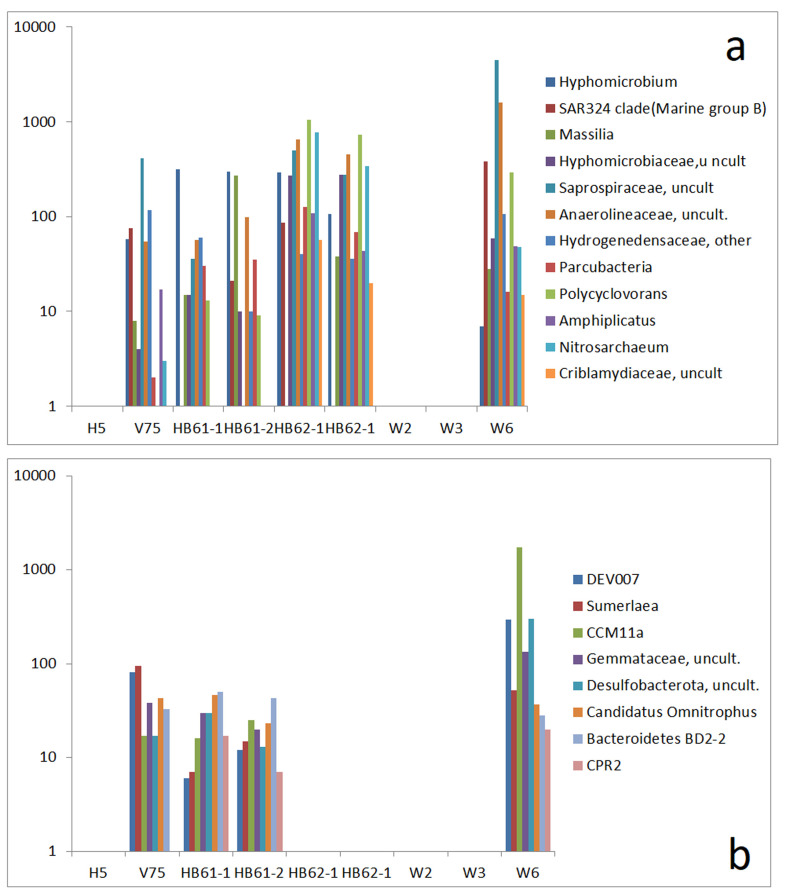
Comparison of absolute read numbers of a set of select OTUs; abundance of OTUs in reference samples and samples W2, W3, and W6; and abundance of OTUs in reference samples and samples W2 and W6 by read numbers: (**a**) OTUs that are present in all reference samples (without H5) and samples W2 and W6 and (**b**) OTUs that are present in W6 and some of the reference samples.

In addition to the above-mentioned groups, we also found a fingerprint-like group of OTUs connecting all reference samples to W2 and W6 and a group connecting the reference samples to W6 only (Figure 7). In the first group, a convincing relationship to marine or other salty environments was not found (Figure 8a). Only the types *PAUC26f* and *JG30-KF-CM66* are related to salty conditions because they are described as sponge-associated bacteria [51,52].

This is in contrast to the second group, which concerns the connection between the reference samples and W6 only. In this group, *Haliangium* [53], *bacteriap 25* [54], *Nitrospira* [55], *NS11-12 marine group* [56], and *SAR202 clade* [57] were described as originating from marine environments. For this group, the similarity between W6 and the samples from saline ash deposit soils (HB61-1, HB61-2, HB62-1, and HB62-2) is striking (Abb. 8b). The relationship between the communities of W6 and those of the saline ash deposit soil samples is supplemented by a further group of OTUs that are absent in H5 (Figure 8a). A small part of the OTUs is described as being related to salty environments, including *Amphiplicatus* [43] and *Polycyclovorans* [58]. This group is complemented by a set of six other OTUs that are only present in V75, HB61-1, HB61-2, and W6, including *Nanobacteria* (Candidate Phyla Radiation group) and *Sumerlaea* [59], a widespread phylum candidate mostly known from deep-sea basins, hot springs, and salt lakes (Figure 8b).

**Figure 9:**
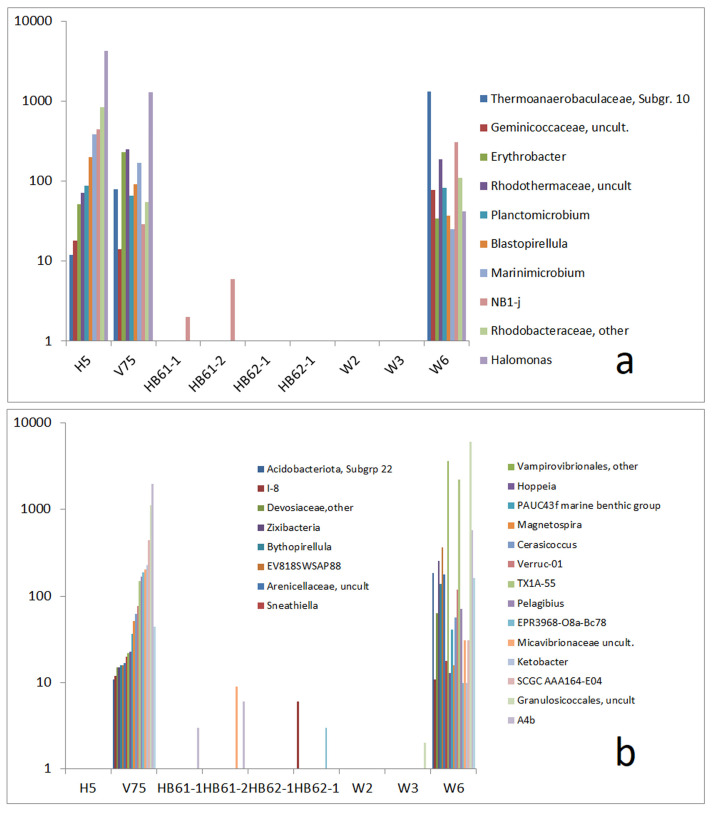
Comparison of absolute read numbers of a set of select OTUs; abundance of OTUs in reference samples and samples W2, W3, and W6 and abundance of OTUs in reference samples and sample W6 by read numbers: (**a**) OTUs that are present in H5, V75, and W6 and (**b**) OTUs that are present in V75 (potash mine dump) and W6 (mine tunnel floor) only.

Finally, two groups of OTUs in W6 are commonly observed in H5 and V75 (Figure 9a) or exclusively in V75 (Figure 9b). Both groups are marked by a significant portion of salty environment-originating types, with mostly ocean-related bacteria such as *Erythrobacter* [60], *Blastopirellula* [61], *Marinimicrobium* [42], and *Halomonas* [62] in the first group and *Hoppeia* [63], *Magnetospira* [64], *Cerasicoccus* [65], *Pelagibus* [66], *Ketobacter* [67], *Bythopirellula* [40] and *Sneathiella* [68] in the second group. These analogies in the composition of the bacterial communities of sample W6, on the one side, and the community of reference samples H5 and V75, on the other side, clearly show that the similarity between the samples is mainly caused by OTUs, mostly at the genus level, which are typically found in marine environments.

From the three samples of the copper mine tunnel, the sample from the bottom (W6) clearly shows the strongest relation to the salt-related community components of the reference samples, whereas the sample from the side wall (W3) displays the weakest relation. Among the reference samples, the soil from the potash mine deposit drainage (V75) is marked by a bacterial community with a lot of salty environment-related components, which have been found in the copper mine too. The relationship between the reference sample from the efflux of the copper mine dump (H5) and the copper mine tunnel samples is weaker than that between the latter and V75. The community components of the reference samples from the saline ash deposits (HB61-1, HB61-2, HB62-1, and HB62-2) show a certain relationship to the samples of the copper mine tunnel too, but they are still weaker and less clearly related to salty environment-adapted bacteria, such as in case of the other two reference samples.

**Figure 10:**
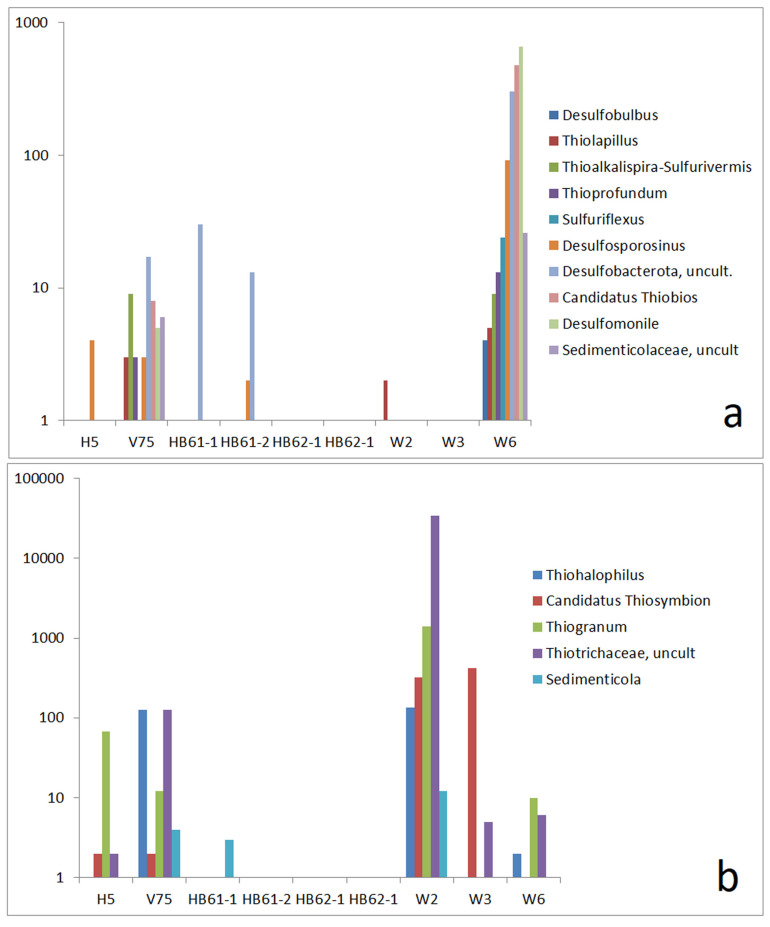
Comparison of absolute read numbers of a set of select OTUs; abundance of OTUs in reference samples and samples W2, W3, and W6 and abundance of selected sulfur-related OTUs by read numbers: (**a**) group of OTUs present in the mine tunnel floor sample W6 and (**b**) OTUs that are preferably present in the copper seam sample W2.

In addition to salt-related OTUs, the abundance of sulfur-related bacteria is noteworthy due to the sulfidic nature of the copper ore. In sample W6, a group of OTUs (Figure 10a) was identified, which is related to sulfur-based metabolisms, including sulfate-reducing bacteria such as *Desulfobulbus* [69], *Desulfosporosinus* [70], and *Desulfomonile* [71], as well as sulfur-oxidizing bacteria such as *Sulfuriflexus* [44], *Thioalkalispira* [72], *Thioprofundum* [73], the NaCl-dependent *Thiolapillus* [74], *Candidatus Thiobios* [75], and an uncultivated strain of *Sedimenticolaceae* belonging to the order *Chromatiales*, which includes sulfide-oxidizing types [76].

In addition to a low read number of *Thiolapillus*, other sulfur-related bacteria are also found in the copper seam sample W2 (Figure 10b). These concern sulfur-oxidizing bacteria like *Thiogranum* [21], *Thiohalophilus* [77], the genus *Sedimenticola* (involving sulfur-oxidizing as well as selenate-reducing species [76,78]), and an uncultivated strain of *Thiotrichaceae*, represented by a very high read number.

**Figure 11:**
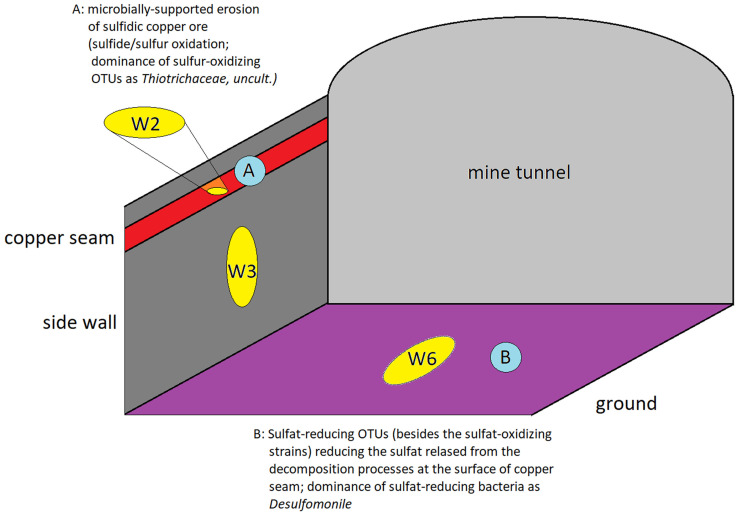
Scheme of sampling sites in the copper mine tunnel: (A) copper seam and (B) floor of the mine tunnel.

The sulfur-related OTUs mentioned above adequately reflect the local situation in the copper mine tunnel (scheme in Figure 11). The surface of the sulfidic copper seam can be attacked by sulfur-oxidizing bacteria. In addition, species that produce elementary sulfur, in particular, sulfate-forming bacteria, were expected to be found there (Figure 11, A). The highly water-soluble sulfate is released and collects on the ground of the mine wall. Thus, sulfate-reducing bacteria can grow in porous sediments on the ground of the mine tunnel (Figure 11, B). This situation corresponds well with the observed types of bacteria, as shown in Figure 11. With *Thiogranum* and, in particular, with the uncultivated *Thiotrichaceae*, a very strong dominance of sulfur-oxidizing strains is observed at the surface of the copper seam (sample W2). Sulfur-related bacteria are present in the sediment of the mine tunnel floor (sample W6) too. However, the read numbers of sulfur-oxidizing bacteria are lower than those in the sample of the copper seam. The sulfur-related part of the bacterial community seems to be dominated by the sulfate-reducers on the floor. A related metabolic way could lead from sulfides via thiosulfate and sulfite to sulfate (S-oxidizers in the sample from the seam; KEGG database, paths 1.8.5.5, 2.8.1.1, and 1.8.3.1) and from sulfate via sulfite back to sulfide (sulfate-reducers in the tunnel sediment sample) [79]. An illustration is given in Supplementary Figure S1.

In principle, significant evidence of the presence of Archaea was expected, but surprisingly, a few archaeal OTUs were found in sample W6 only. The absence or low abundance of Archaea in the investigated samples is in contrast to former investigations of soil material taken from archaeological places and from aboveground samples from ancient copper mining areas [80]. Sample W6 showed 1612 reads (0.95% of all reads) for Cand. Nitrosopumilus and 48 reads (0.03% of all reads) for Nitrosarchaeum. Both OTUs belong to Crenarchaeota, Fam. Nitrosopumilaceae. Nitrosarchaeum is an ammonia-oxidizing microorganism and was first isolated from agricultural soil [81].

Due to the known high salinity of aqueous phases in the mine tunnel, reflected by the very high electrical conductivity of samples, it is expected that all growing bacteria are halophilic or at least highly salt-tolerant. This is largely confirmed by the numerous identified genera, which are described in the literature as halophilic or salt-tolerant. The sulfur-related bacteria discussed above should also have this halophilic or salt-tolerant characteristic. The example of the copper mine shows that such a special environment could be interesting for searching for extremophiles with special metabolic properties.

## 4. Conclusions

The investigations reveal that disused copper mines, such as the one in Wettelrode, are fascinating sites for the development of extremophilic bacteria, particularly halophiles. Notably, samples from the copper seam and the mine tunnel floor contained substantial numbers of halophilic bacteria, with both locations partially characterized by site-specific types. The presence and abundance of many halophilic genera (or other OTUs) show similarities to samples from the drainage of a disused industrial copper mine tailings dump and the efflux of an aboveground potash mine deposit. To a lesser extent, analogies are also observed with archaeologically excavated soil samples from a preindustrial saline ash deposit.

The distinctive nature of the observed bacterial communities is not only shaped by the highly saline environment but is also significantly influenced by the sulfidic composition of the copper ore. This is reflected in the overall high prevalence of bacteria with sulfur-related metabolisms, as well as in variations in the bacterial communities depending on the specific conditions of the sampling sites. In particular, the surface of the copper seam was found to be dominated by sulfur-oxidizing strains.

## Data Availability

Data are available upon request to the authors.

## Supplementary Materials

The following supporting information can be downloaded at: https://www.mdpi.com/article/10.3390/life15020204/s1.
