# Supplementary material for: Bacterial Communities from the Copper Mine of Wettelrode (Germany)
Source: Life (Basel). 2025 Jan 29;15(2):204. doi: 10.3390/life15020204 (PMC11856635; doi:10.3390/life15020204)
Supplement: Supplementary file 1 [file life-15-00204-s001.zip › life-3354059-supplementary.pdf]

Supplementary Table S1:

Examples of comparatively low electrical conductivities observed in top soil samples from natural environment

Place	internal lab No	character	electrical conductivity
Königsee, Stadtwald	V24	forest	29.3 µS/cm
Halle/S. , Dölauer Heide	T88	forest	32.6 µS/cm
Sondershausen, Hainleite	V42	forest	43.1 µS/cm
Kaltenlengsfeld	T30	forest	54.1 µS/cm
Hümpfershausen, Roßberg	V12	forest	57.8 µS/cm
Rastenberg, Streitholz	T85	forest	64.7 µS/cm
Mäbendorf	T52	forest	66.5 µS/cm
Aschenhausen, Diesburg	T17	forest	95.9 µS/cm

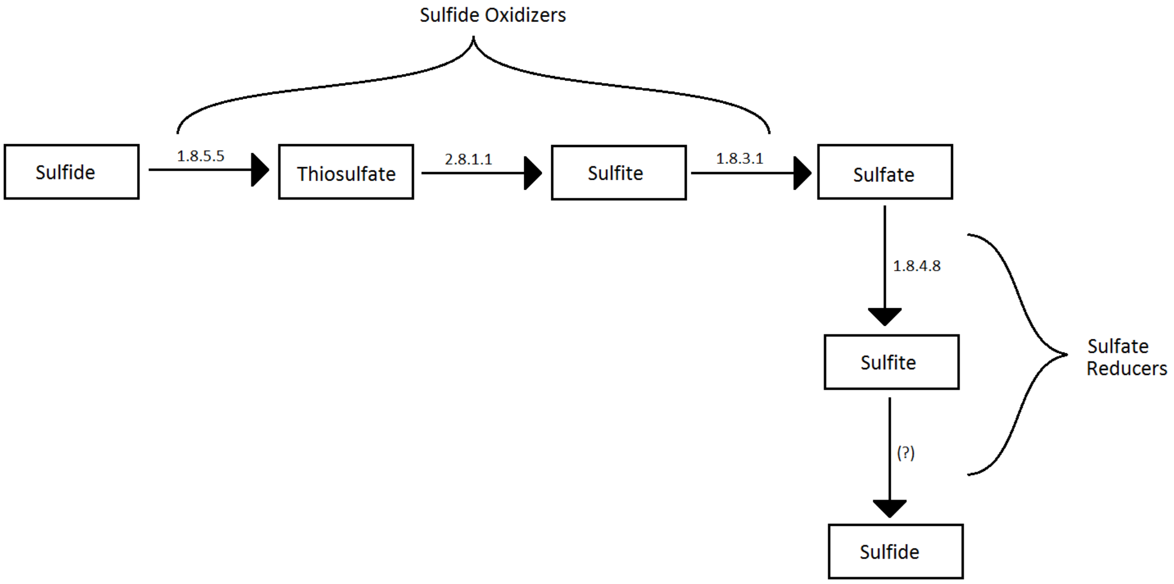

Supplementary figure S1

Possible metabolic pathways in Sulfid oxidation and sulfat reduction (KEEG database [80])
